# Prevalence and Subtypes of Mild Cognitive Impairment in Parkinson’s Disease

**DOI:** 10.1038/srep33929

**Published:** 2016-09-21

**Authors:** Blake J. Lawrence, Natalie Gasson, Andrea M. Loftus

**Affiliations:** 1 Curtin Neuroscience Laboratory, School of Psychology and Speech Pathology, Curtin University, Kent Street, Bentley, Western Australia, 6102, Australia; 2 ParkC Collaborative Research Group, Curtin University, Kent Street, Bentley, Western Australia, 6102, Australia

## Abstract

The current study examined the prevalence and subtypes of Mild Cognitive Impairment (MCI) in an Australian sample of people with Parkinson’s Disease (PD). Seventy participants with PD completed neuropsychological assessments of their cognitive performance, using MDS Task Force Level II diagnostic criteria for PD-MCI. A cut-off score of less than one standard deviation (SD) below normative data determined impaired performance on a neuropsychological test. Of 70 participants, 45 (64%) met Level II diagnostic criteria for PD-MCI. Among those with PD-MCI, 42 (93%) were identified as having multiple domain impairment (28 as amnestic multiple domain and 14 as nonamnestic multiple domain). Single domain impairment was less frequent (2 amnestic/1 nonamnestic). Significant differences were found between the PD-MCI and Normal Cognition groups, across all cognitive domains. Multiple domain cognitive impairment was more frequent than single domain impairment in an Australian sample of people with PD. However, PD-MCI is heterogeneous and current prevalence and subtyping statistics may be an artifact of variable application methods of the criteria (e.g., cut off scores and number of tests). Future longitudinal studies refining the criteria will assist with subtyping the progression of PD-MCI, while identifying individuals who may benefit from pharmacological and nonpharmacological interventions.

Parkinson’s disease (PD) is now understood as a multifaceted neurodegenerative disorder with heterogeneous motor and non-motor symptoms^1^. Approximately 30% of people with PD demonstrate cognitive impairment and up to 50% of those progress to PD-Dementia after more than 10 years of disease duration^2^,^3^. Cognitive impairments in PD comprise four subtypes; amnestic single, amnestic multiple, nonamnestic single and nonamnestic multiple. The four subtypes reflect deficits across five cognitive domains, including: memory, attention/working memory, language, visuospatial, and executive functions^4^,^5^.

Several biological and epidemiological risk factors are associated with cognitive deficits in PD, with some studies reporting cognitive impairment is present even at time of diagnosis^6^,^7^. To standardize assessment, the Movement Disorder Society (MDS) Task Force developed new diagnostic criteria for PD-Mild Cognitive Impairment (PD-MCI)^8^. Preceding the criteria, most studies adopted the method proposed by Petersen *et al*.^9^ which specifies a decline in memory. PD-MCI is, however, heterogeneous and many people demonstrate impairments across the spectrum of cognitive domains^10^. Recent studies adopting the new MDS diagnostic criteria report variable results^11^,^12^. These studies also applied varying diagnostic cut off scores and weighting of tests per cognitive domain, which may influence the reported prevalence of cognitive impairment in PD.

The high prevalence and significant impact of cognitive impairment on quality of life for people with PD^13^, indicate that any standardised criteria developed for international use needs to be validated and examined across multiple populations of PD. To date, no study has applied the MDS criteria for PD-MCI to an Australian sample. This study provided a novel application of the MDS Task Force PD-MCI Level II diagnostic criteria to an Australian sample of people with PD. This study also examined PD-MCI frequency differentials at varying diagnostic cut off scores to explore subtype classifications and advance our understanding of cognitive impairments in PD.

## Results

Seventy participants completed neuropsychological assessments, with 64.3% (*N* = 45) classified as ‘PD-MCI’ and the remaining participants classified as ‘PD-Normal Cognition (PD-NC)’ (*N* = 25). For demographic variables, there were no statistically significant group differences. There were significant differences between groups on all cognitive outcomes (excluding SOC). Table 1 provides demographic and neuropsychological test results for participants in the PD-MCI and PD-NC groups.

Internal reliability varied between adequate (0.40 to 0.50) to excellent (>0.90) and was computed for 10 outcomes: UPDRS-II (α = 0.80) TISC (KR-20 = 0.47), MMSE (KR-20 = 0.54); PD-CRS (α = 0.84), LNS (KR-20 = 0.92), Stroop (Colour-Word) Test (KR-20 = 0.96), BNT (KR-20 = 0.54), Similarities (α = 0.68), JLO (KR-20 = 0.90) and HVLT (KR-20 = 0.78). Low internal reliability scores were identified for the TISC, MMSE, BNT and Similarities outcomes. However, due to the diversity of cognitive constructs, using cut off scores for Cronbach’s α may subtract from the scale’s primary purpose in the context of the research^14^. All outcomes were therefore reported in this study and the current authors suggest interpreting the outcomes with low reliability with caution.

### PD-MCI subtypes according to MDS criteria

**Figure 1:**
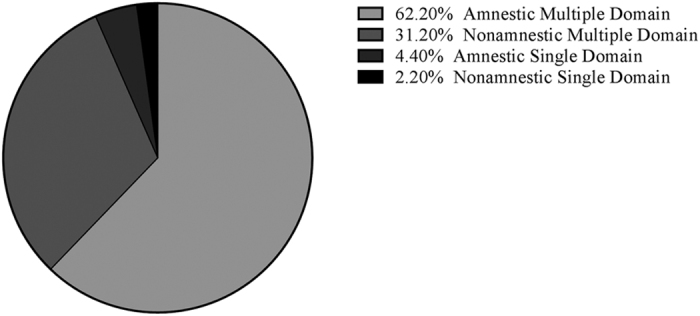
Distribution of PD-MCI subtypes using a one standard deviation cut off.

Among participants who met the MDS Task Force criteria for PD-MCI (*N* = 45), 93.4% demonstrated multiple domain impairment and only 6.6% showing single domain impairment (4.4% memory and 2.2% visuospatial; Fig. 1).

**Figure 2:**
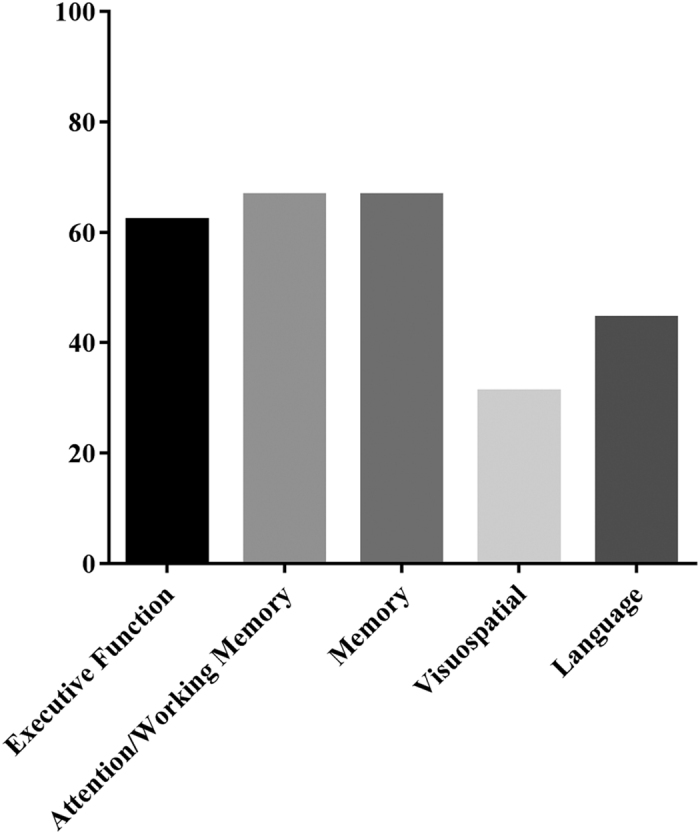
Percentage of participants with cognitive impairment by domain when using a one standard deviation cut off.

Cognitive deficits were heterogeneous among participants with multiple domain PD-MCI. In total, 62.2% (*N* = 28) of participants were classified as amnestic multiple domain with 11 different patterns of impairments identified (Table 2). Moreover, 31.2% (*N* = 14) of participants were classified as nonamnestic multiple domain and nine different patterns of impairments identified. When comparing individual cognitive domains for all PD-MCI (Fig. 2), executive function was impaired in 62.2% (*N* = 28) of participants, attention/working memory in 66.7% (*N* = 30), memory in 66.7% (*N* = 30), visuospatial in 31.2% (*N* = 14) and language in 44.4% (*N* = 20).

### Post-hoc analyses

Following the high frequency of PD-MCI (64.3%) when using a 1 SD cut-off below normative data, post-hoc analyses were conducted to examine whether using a 2 SD cut off would result in frequency differentials (Table 3). The frequency of PD-MCI decreased from 64.3% to 28.6% (*N* = 20). Among participants with PD-MCI, however, the frequency of subtype classifications remained relatively stable. Overall, 90% (*N* = 18) of participants with PD-MCI demonstrated multiple domain impairment and only 10% showed single domain impairment (*N* = 1 for memory and *N* = 1 for attention/working memory). Amnestic multiple domain remained most frequent (*N* = 10, 50%) with five different patterns of impairments, followed by nonamnestic multiple domain (*N* = 8, 40%) with five different patterns of impairments. Both amnestic single and nonamnestic single domains showed the least frequency of impairment (*N* = 1, 5% individually). Following the 2 SD cut off, executive function was impaired in 75% (*N* = 15) of participants, attention/working memory in 45% (*N* = 9), memory in 50% (*N* = 10), visuospatial in 45% (*N* = 9) and language in 10% (*N* = 2).

## Discussion

This study was the first application of MDS Task Force criteria for PD-MCI in an Australian sample. Using the criteria at the 1 SD cut off score, 64.3% of participants were diagnosed as PD-MCI. Among those with PD-MCI, 93.4% presented with multiple domain impairments (i.e., deficit test results in more than one cognitive domain), and 6.6% with single domain impairment. Attention/working memory, executive function, and memory impairments were the most frequently impaired cognitive domains. Language and visuospatial domains demonstrated less impairment. These results support those of Cholerton *et al*.^12^ and Goldman *et al*.^10^ who found that 63–67% of their samples had PD-MCI and that 91.5–95% of those participants had multiple domain impairments. Marras *et al*.^11^ reported that 93% of their sample with PD-MCI had multiple domain impairment, despite an overall PD-MCI prevalence of only 33%. Recent application of the new criteria also revealed that attention/working memory, executive function and memory domains were most frequently impaired in PD-MCI^10^,^12^. These results, however, conflict with prevalence statistics preceding the new diagnostic criteria. Studies predating the criteria indicate a significantly lower prevalence (19% to 38%) of PD-MCI and some studies identified single domain impairment as more common than multiple domain impairment^2^,^15^,^16^.

Several reasons have been proposed for the varying frequency of PD-MCI across studies. Compared to previous methods, the new MDS criteria is less stringent when diagnosing multiple domain (i.e., impairment on one test per domain) compared to single domain (i.e., impairment on two tests in one domain) subtypes, which will invariably identify more people with multiple domain impairment^10^. Introducing a more conservative criterion for the multiple domain subtype (e.g., impairment on two tests per domain) will likely reduce the biased frequency of multiple domain impairment. In addition, several verbal memory, visuospatial, and attention tests have demonstrated appropriate diagnostic specificity for PD-MCI^17^, and administering these tests in future research may provide a more accurate estimate of the multiple domain subtype.

In studies preceding the MDS Task Force criteria, variable use of SD cut offs increased the heterogeneity of the frequency of PD-MCI and this issue is yet to be resolved^18^. The new diagnostic criteria suggest 1 to 2 SD cut offs for establishing cognitive impairment with normative data^8^, but Liepelt-Scarfone *et al*.^18^ have shown that PD-MCI diagnoses vary between 56.4% (using <1 SD) and 9.9% (using <2 SDs). Having said this, a recent study identified 2 SDs as the most sensitive and specific cut off for diagnosing PD-MCI using the new criteria^10^. Using a 2 SD cut off in the current study reduced the frequency of PD-MCI from 64.3% (using 1 SD) to 28.6%, but the frequency of subtype classifications remained relatively stable (i.e., multiple domain impairment remained more frequent than single domain). Language impairment, however, reduced from 44.4% (*N* = 20) using a 1 SD cut off to 10% (*N* = 2) using a 2 SD cut off. Compared to other cognitive domains, this result suggests that language impairment may be less frequent in PD-MCI. Impairment across all cognitive domains was prevalent among 11.1% (*N* = 5) of participants using a 1 SD cut off, but this reduced to nil participants using a 2 SD cut off. This finding supports the current characterisation of PD-MCI, with most individuals demonstrating impairment within multiple, but not all, cognitive domains (e.g., executive function and memory)^12^. Using a 1 SD cut off may, however, be too liberal and not sufficiently specific for identification of PD-MCI subtypes^10^. Overall, the reduction in the frequency of PD-MCI is similar to previous prevalence estimates that adopted more conservative 1.5 SD^11^ and 2 SD^19^ cut off scores. The MDS Task Force, however, suggest using a 1 SD cut off to detect impaired cognition in higher functioning individuals, who may have noticed a decline in their cognitive functioning but do not meet the stricter criteria of 1.5 to 2 SDs^8^.

The inconsistent use of, and weighting of, cognitive tests per domain may also bias diagnosis and subtyping of PD-MCI. The MDS Task Force recommends two tests per cognitive domain to ensure consistency across studies and the reliable external validity of results^8^. Recent studies adopting the criteria have used between 3 and 7 tests/subtests per domain, which is more than recommended^10^,^12^. Inclusion of more tests in any one domain increases the risk of a Type I error and may falsely inflate the prevalence of PD-MCI^20^. A recent study showed that when using MDS Task Force recommendations (10 or more neuropsychological tests), approximately 13% of people with PD and normal cognition will demonstrate impaired performance on two or more tests^20^. The recent increase in prevalence of PD-MCI may, therefore, be associated with the inclusion of more neuropsychological tests in the assessment of PD-MCI, which may lead to more false-positive diagnoses. As previously noted, a more conservative use of tests (e.g., impairment on two tests per domain) when diagnosing multiple domain PD-MCI and applying a more stringent cut off score (e.g., <2 SDs below normative data) may reduce the risk of Type 1 errors in research and clinical settings. While acknowledging these issues, further refinement of the PD-MCI criteria will determine the ideal classification method, appropriate cut off scores, and the optimal number and selection of tests for diagnosis.

Although recent studies have used varying cut off scores, subtype classifications in this study are consistent with recent findings^21^. Most participants were identified as multiple domain PD-MCI, which comprised 20 different combinations of impaired domains. Cholerton *et al*.^12^ also reported 19 combinations of impaired domains within their multiple domain subtype. Although this may be an artefact of the MDS diagnostic criteria (i.e., 1 SD cut off has shown low specificity^10^), this heterogeneous distribution across multiple domains is a hallmark feature of PD-MCI^4^. Research has identified diverse pathophysiological changes and characteristics that may underlie the heterogeneous presentation of PD-MCI^3^.

Most participants in this study demonstrated memory and executive function impairments, but there were considerable concomitant deficits across domains. The variability of PD-MCI has been associated with protein/neurotransmitter abnormalities and genetic characteristics^3^. Catecholaminergic changes involving frontostriatal dopaminergic deficits are associated with executive function impairment, and deficiency of acetylcholine is associated with impaired posterior cortical function of memory, language and visuospatial abilities^3^,^7^,^22^. Alpha-synuclein infiltration (as Lewy based pathology) of the limbic system and neocortex has also been associated with amnestic cognitive impairment in PD^23^. The range of neurotransmitter changes demonstrate the complex pathology of different cognitive impairments in PD.

Kehagia *et al*.^4^ suggest that genetic characteristics may account for patterns of decline in PD-MCI. The ‘dual syndrome hypothesis’ proposes two distinct genetic syndromes (executive and posterior cortical) that affect executive function and memory/visuospatial abilities in PD, and often present early in the disease^4^. A recent study tested this hypothesis and found associations between a genetic variation (rs4680 polymorphism of the COMT gene) which modulated executive function and two genetic variations (APOE allelic and MAPT haplotype) which independently modulated posterior cortical functions of memory and visuospatial abilities, respectively^24^. These studies provide initial evidence that frontal or posterior cortical cognitive deficits are associated with specific genetic and neurotransmitter abnormalities. Neuroimaging was beyond the scope of the present study, but the heterogeneity of multiple domain PD-MCI in this study does not support the ‘dual syndrome hypothesis’ (e.g., participants demonstrated many patterns of impairment across all cognitive domains). Having said this, research shows considerable overlap between the executive and posterior cortical syndromes and further clinical trials combining neuroimaging and neuropsychological testing are required^25^.

Participants with PD-MCI performed significantly worse (compared to participants with PD-NC) across all cognitive domains, including global cognition. Similar results were reported by Goldman *et al*.^10^ and Marras *et al*.^11^ In both of these studies, PD-MCI groups performed significantly worse on cognitive outcomes compared to the unimpaired groups. Group allocation was determined by cognitive performance, and as such, significant differences between group scores were to be expected. In this study, however, a conflicting result was reported for executive function. Compared to the Controlled Oral Word Association Test (COWAT), scores on the Stockings of Cambridge (SOC) test demonstrated no difference between groups, indicating comparative performance between those with and without PD-MCI. A recent systematic review and meta-analysis highlighted the multifaceted nature of executive function and the challenges faced when researching this cognitive domain in PD^26^. Executive function is often referred to as an ‘umbrella’ concept used to describe many subcomponent abilities, including purposive action (execution), volition, planning, effective performance, attentional control, set-shifting, inhibition and managing behaviour^27^,^28^,^29^. Consequently, individual neuropsychological tests are often unable to capture and measure the full spectrum of executive function. Predominantly, the SOC test involves rule learning, planning and execution, whereas the COWAT requires set shifting (between trials) and attentional control. In addition, studies have shown separation of ‘hot’ and ‘cold’ executive function abilities. ‘Cold’ cognitive tasks are described as neutrally affective and involve cognitive flexibility, while ‘hot’ cognitive tasks are influenced by emotion and motivated reasoning^30^. Due to the complexity of executive function and the inherent specificity of neuropsychological tests, people with PD may show impaired performance on individual tests which do not represent impairment across the entire domain^26^. Therefore it is important that the exact tests used for diagnosis are standardised.

When examining demographic variables, there were no significant differences between groups. Participants in the PD-MCI group were slightly older and had slightly less years of education, but these differences were not significant. Recent studies have reported no educational difference between people with and without cognitive impairments in PD^10^,^11^,^12^,^15^. Some studies, however, reported older age and less years of education associated with cognitive decline in PD^7^,^31^,^32^. These conflicting results suggest future longitudinal research is required to determine the long-term relationship between years of education and cognitive impairment in PD. Due to participant burden, motor symptom severity was not directly measured in the current study. Levodopa equivalent dose and disease duration were measured and the findings indicate no group differences, which suggests severity of motor symptoms were similar across groups^33^.

### Limitations and recommendations for future research

The main limitation of this study was the cross sectional design, which involved only baseline cognitive assessments. Collecting data at one time-point limits examination of which neuropsychological tests are most appropriate, and of which domains of impairment are most predictive of cognitive decline in PD. This sample had relatively high educational levels and low years of disease duration. These characteristics are comparable to cohorts from recent studies^11^,^12^, but may limit the generalisability of the results to the wider PD population. Lastly, there was low internal consistency for some cognitive measures, which must be noted when interpreting the results.

Future studies should adopt longitudinal designs to validate and provide suggestions for the refinement of the MDS Task Force diagnostic criteria for PD-MCI. Future studies need to determine which neuropsychological tests are most reliable and valid over time, the most appropriate number of tests per cognitive domain (to control inflation of Type I errors), and the most sensitive and specific cut off scores for diagnostic purposes. The MDS criteria also needs to be applied to different age groups with varying degrees of cognitive impairment, disease severity and cognitive reserve (educational/occupational attainment). Recent and ongoing longitudinal studies are examining biomarker, epidemiological and neuropsychological risk factors associated with cognitive decline in PD^7^,^20^,^24^,^32^,^34^. However, future studies need to adopt an interdisciplinary approach by integrating clinical neuroscience, neuroimaging and neurobiology with the MDS criteria, to provide a greater understanding of PD-MCI. Moreover, studies must be transparent in their reporting of the normative datasets used to establish diagnoses of PD-MCI. As explained by Strauss *et al*.^35^, selection of appropriate normative data is equally as important as choosing a reliable and valid neuropsychological test. Using a normative dataset that is not a demographical match to a participant’s characteristics is problematic, given that norm-referenced scores are directly related to clinical and research consequences such as prevalence rates, diagnosis, and pharmacological/nonpharmacological interventions^36^.

The etiology and profile of PD-MCI is heterogeneous, with some people reverting back to normal cognition and many others progressing to PD-Dementia^6^. There is no current therapeutic intervention to halt or delay cognitive decline in PD^37^. Clinical trials are examining the potential of pharmacological treatments, but two recent studies found no improvements in cognition^38^,^39^. The limited empirical support of pharmacological treatment for PD-MCI has led to an increase in research assessing nonpharmacological interventions for cognition in PD^40^. Specifically, cognitive training and non-invasive brain stimulation have improved cognition in PD^41^,^42^. However, most studies included participants without cognitive impairment and significant methodological heterogeneity limits the reliability of results^37^. Despite current limitations, nonpharmacological interventions may be a therapeutic alternative for people with PD-MCI who are already burdened by dopaminergic medications.

When applying the MDS Task Force diagnostic criteria for PD-MCI, this study found 64% of participants (using a 1 SD cut off) were cognitively impaired (i.e., demonstrated PD-MCI), and this figure reduced to 28% with PD-MCI when using a 2 SD cut off. Despite the change in frequency of impairments, most participants with PD-MCI were classified as multiple domain subtype which is consistent with recent findings^21^. Although further validation and refinement of the diagnostic criteria is required, the significant prevalence and heterogeneous nature of PD-MCI is well documented. Future studies need to integrate the MDS criteria with longitudinal designs of interdisciplinary research, to identify subtypes of PD-MCI and individuals most likely to develop PD-Dementia. Subtyping PD-MCI may also allow researchers to determine which subtypes will benefit from pharmacological and nonpharmacological interventions that may delay or halt progression of cognitive decline.

## Method

This study used a cross-sectional design to measure cognition in people with PD, using the MDS diagnostic criteria for PD-MCI^8^. Neuropsychological assessments were completed at Curtin University’s Neuroscience Laboratory between March and September, 2015.

### Participants

Participants were adults with PD living in Western Australia. The following inclusion criteria were used: (1) diagnosed with idiopathic PD by a neurologist or geriatrician in accordance with the United Kingdom Parkinson’s Disease Society Brain Bank Clinical Diagnostic Criteria, (2) a stable response to antiparkinsonian medication for a minimum period of 2 months, and (3) cognitive deficits that do not interfere with functional independence. Exclusion criterion was presence of PD-Dementia.

### Neuropsychological assessment

Neuropsychological assessments were conducted in two phases. Participants were first screened over the telephone for the presence of dementia (using the TISC-30)^43^. Participants then completed an extensive neuropsychological assessment at Curtin University. In accordance with the MDS Task Force Level II diagnostic criteria for PD-MCI, two measures were selected to assess each of the five cognitive domains impacted in PD-MCI^8^. The following measures have been recommended by the MDS Task Force for use in PD and were used to assess functioning. *Executive function* was assessed using the Stockings of Cambridge (SOC) subtest from the Cambridge Neuropsychological Test of Automated Batteries (CANTAB^TM^) and the phonemic verbal fluency subtest of the Controlled Oral Word Association Task (COWAT)^44^. *Attention and working memory* was assessed using the Letter-Number Sequencing (LNS) subtest from the Wechsler Adult Intelligence Scale-IV (WAIS-IV)^45^ and the Stroop (Colour-Word) Test^46^. *Memory* was assessed using the Hopkins Verbal Learning Test-Revised (HVLT-R)^47^ immediate recall subtest and the Paragraph Recall subtest of the Rivermead Behavioural Memory Test (RMBT)^48^. *Visuospatial abilities* were assessed using the Judgement of Line Orientation (JLO) test^49^ and the Hooper Visual Organisation Test (HVOT)^50^. *Language* was assessed using the Boston Naming Test-Short Form (BNT-Short Form)^51^ and the Similarities subtest from the WAIS-IV battery^45^.

Global cognition was assessed using the Parkinson’s Disease – Cognitive Rating Scale (PD-CRS)^52^ and the Mini Mental State Examination (MMSE)^53^. Premorbid intelligence and activities of daily living were assessed by the Australian version of the National Adult Reading Test (AUSNART)^54^ and Unified Parkinson’s Disease Rating Scale (section II)^55^, respectively. A UPDRS-II^55^ summary index score greater than 3 was used to exclude participants with impaired activities of daily living (i.e., cognitive deficits that significantly impact functional independence). Demographic information including age, disease duration (years) and current daily levodopa dopaminergic medication were also collected^56^. This study was approved by Curtin University’s Research Ethics Committee prior to contact with participants (approval number: HR 189/2014). All research methods were carried out in accordance with ethics committee guidelines and all participants provided informed consent prior to completing neuropsychological assessments. All assessments were conducted during participants’ ‘ON’ stage of medication use.

### Cognitive diagnosis

Following each assessment, results were scored and interpreted using standardised normative data from healthy older adults (Supplementary Table 1). PD-MCI was diagnosed as less than one standard deviation (SD) below normative scores on two or more neuropsychological tests^8^. The MDS Task Force suggest the use of 1 to 2 SD cut offs below normative scores. Raw scores were used to determine impairment on all tests, excluding the LNS and Similarities tests. The LNS and Similarities raw scores were converted into scaled scores (as per WAIS-IV instructions), and then compared to normative data^45^. In accordance with the MDS criteria^8^, a cut off of 1 SD was used in this study to accommodate the likelihood that the community based cohort may include higher functioning adults living independently, who may not report cognitive deficits but demonstrate impairment during formal neuropsychological assessment. Also, subjective report of cognitive decline has shown low accuracy in PD^57^. Therefore, individuals in this study were not required to meet the criteria of reporting cognitive decline. For participants who met the Level II criteria, the following PD-MCI subtype classifications were applied: (1) amnestic single domain (impairment on two memory tests); (2) nonamnestic single domain (impairment on two or more non-memory tests); (3) amnestic multiple domain (impairment on two or more tests, including memory) and (4) nonamnestic multiple domain (impairment on two or more tests, not including memory). Participants who did not meet the Level II criteria were classified as having ‘Normal Cognition’.

### Statistical analysis

Statistical Package for the Social Sciences (SPSS) 22.0 was used for statistical analyses. Descriptive statistics for demographic data and neuropsychological test scores were computed, and frequency estimates were calculated to describe the prevalence of cognitive impairment and PD-MCI subtypes. Independent samples *t* tests and a Mann-Whitney *U* test (for a non-parametric outcome) were used to examine if there were statistically significant differences between the ‘PD-MCI’ and ‘Normal Cognition’ (i.e., those without PD-MCI) groups on demographic variables and neuropsychological outcomes^58^,^59^. An alpha level of 0.05 was applied to demographic variables and a Bonferroni-adjusted alpha level was applied to cognitive outcomes, where there were multiple comparisons per domain (i.e., *p* < 0.025). Where possible, the internal reliability of outcomes were computed using two methods: (1) the Kuder-Richardson 20 (KR-20), and (2) Cronbach’s α^60^,^61^. Both methods produce estimates of internal consistency, though the KR-20 assesses tests with dichotomous response items (e.g., ‘correct’ or ‘incorrect’) and Cronbach’s α examines tests with any response scale^62^. For cognitive tests, internal consistency of ≥0.70 is acceptable for research purposes^59^.

## Additional Information

**How to cite this article**: Lawrence, B. J. *et al*. Prevalence and Subtypes of Mild Cognitive Impairment in Parkinson’s Disease. *Sci. Rep.* **6**, 33929; doi: 10.1038/srep33929 (2016).

## Supplementary Material

Supplementary Information

## Figures and Tables

**Table 1:** Comparison of demographic and neuropsychological test scores for PD-MCI and Normal Cognition groups.

Domain	Outcome	PD-MCI (*N* = 45)		NC (*N* = 25)		Diff. of means	
		*M*	*SD*	*M*	*SD*	*t*	*p*
	Gender (% male)	62.2% (*N* = 28)	64% (*N* = 16)	—	0.88^+^		
	Age^++^	68.53	9.92	64.12	7.10	−1.96	0.05
	Education^++^	13.60	3.10	14.52	2.87	1.22	0.23
	Premorbid IQ	106.97	8.01	106.81	21.71	-0.04	0.97
	Disease Duration^++^	5.81	4.58	5.90	4.99	0.08	0.94
	LED	398.43	350.33	335.19	254.15	-0.79	0.43
	UPDRS-II	1.08	0.62	0.89	0.54	−1.30	0.20
Global	TISC	22.42	2.95	24.48	2.47	2.96	0.004*
	MMSE	25.56	2.95	27.84	1.62	3.57^×^	0.001*
	PD-CRS	81.07	19.48	100.28	12.10	4.47^×^	0.001**
EF	COWAT	32.24	15.01	45.80	12.36	3.85	0.001**
	SOC	6.22	2.08	7.24	2.06	1.96	0.60
Atten.WM	LNS (SS)	8.36	3.64	11.56	2.16	4.02	0.001**
	LNS (RS)	16.09	5.88	20.52	2.43	3.59^×^	0.001*
	Stroop Test	24.51	12.19	38.24	10.05	4.80	0.001**
Memory	HVLT	21.60	6.82	28.80	5.48	4.53	0.001**
	Paragraph Recall	4.56	2.31	7.08	1.79	4.71	0.001**
Language	BNT	13.27	1.66	14.32	0.80	2.98^×^	0.001**
	Similarities (SS)	8.76	1.88	10.84	1.52	4.74	0.001**
	Similarities (RS)	21.09	3.97	26.40	2.75	5.93	0.001**
VS	JLO	21.29	7.62	26.64	3.49	3.31^×^	0.001*
	HVOT	22.11	3.96	25.24	2.10	4.32^×^	0.001**

PD-MCI = Parkinson’s Disease-Mild Cognitive Impairment; NC = Normal Cognition; *M* = mean; *SD* = standard deviation; *t* = t-statistic; *p* = alpha level of significance; ^+^ = non-parametric Mann-Whitney *U* test; ^++^ = years; ^×^ = equal variances not assumed; * = *p* < 0.05; ** = *p* < 0.001; Global = global cognition; EF = executive function; Atten./WM = Attention/working memory; VS = visuospatial; LED = levodopa equivalent dose; UPDRS-II = Unified Parkinson’s Disease Rating Scale-section 2 (activities of daily living); TISC = Telephone Interview for Cognitive Status; MMSE = Mini-Mental State Examination; PD-CRS = Parkinson’s Disease-Cognitive Rating Scale; COWAT = Controlled Oral Word Association Test; SOC = Stockings of Cambridge; LNS = Letter-Number Sequencing; SS = Scaled score; RS; Raw score; HVLT = Hopkin’s Verbal Learning Test; BNT = Boston Naming Test; JLO = Judgement of Line Orientation; HVOT = Hooper’s Visual Organisation Test.

**Table 2:** Distribution of PD-MCI subtypes and domain impairments using a one standard deviation cut off score.

PD-MCI Subtype	Domains Impaired	*N* (%)
Amnestic Multiple	All domains	5 (11.1)
	Memory + EF	5 (11.1)
	Memory + Attention/WM	5 (11.1)
	Memory + EF + Attention/WM	3 (6.7)
	Memory + Attention/WM + Language	3 (6.7)
	Memory + EF + Attention/WM + Language	2 (4.4)
	Memory + EF + Attention/WM + Visuospatial	1 (2.2)
	Memory + Attention/WM + Visuospatial	1 (2.2)
	Memory + Language	1 (2.2)
	Memory + EF + Language	1 (2.2)
	Memory + Language + Visuospatial	1 (2.2)
	Subtotal	28 (62.2)
Nonamnestic Multiple	EF + Attention/WM	4 (8.8)
	EF + Language	3 (6.7)
	EF + Visuospatial	1 (2.2)
	EF + Attention/WM + Language	1 (2.2)
	EF + Attention/WM + Language + Visuospatial	1 (2.2)
	EF + Attention/WM + Visuospatial	1 (2.2)
	Attention/WM + Language	1 (2.2)
	Attention/WM + Visuospatial	1 (2.2)
	Attention/WM + Language + Visuospatial	1 (2.2)
	Subtotal	14 (31.2)
Amnestic Single	Memory	2 (4.4)
Nonamnestic Single	Visuospatial	1 (2.2)
	Total	45 (100)

EF = Executive Function; Attention/WM = Attention/Working Memory.

**Table 3:** Distribution of PD-MCI subtypes and domain impairments using a two standard deviation cut off score.

PD-MCI Subtype	Domains Impaired	*N* (%)
Amnestic Multiple	Memory + EF	4 (20)
	Memory + EF + Attention/WM + Visuospatial	3 (15)
	Memory + EF + Attention/WM	1 (5)
	Memory + Attention/WM	1 (5)
	Memory + Language	1 (5)
	Subtotal	10 (50)
Nonamnestic Multiple	EF + Visuospatial	4 (20)
	EF + Attention/WM	1 (5)
	EF + Language	1 (5)
	EF + Attention/WM + Visuospatial	1 (5)
	Attention/WM + Visuospatial	1 (5)
	Subtotal	8 (40)
Amnestic Single	Memory	1 (5)
Nonamnestic Single	Attention/WM	1 (5)
	Total	20 (100)

EF = Executive Function; Attention/WM = Attention/Working Memory.
