# Supplementary material for: Prevalence and Subtypes of Mild Cognitive Impairment in Parkinson’s Disease
Source: Sci Rep. 2016 Sep 21;6:33929. doi: 10.1038/srep33929 (PMC5030649; doi:10.1038/srep33929)
Supplement: Supplementary Information [file srep33929-s1.pdf]

Prevalence and Subtypes of Mild Cognitive Impairment in Parkinson's Disease

Blake J. Lawrence[1,2]*, Natalie Gasson[1,2], and Andrea M. Loftus[1,2]

[1] Curtin Neuroscience Laboratory, School of Psychology and Speech Pathology, Curtin University, Kent Street, Bentley, Western Australia, 6102, Australia

[2] ParkC Collaborative Research Group, Curtin University, Kent Street, Bentley, Western Australia, 6102, Australia

*blake.lawrence@postgrad.curtin.edu.au

Supplementary Table 1. Sources of normative data for neuropsychological tests.

Cognitive Domain	Neuropsychological Test	Norm Reference
Executive Function	Stockings of Cambridge	Cambridge Neuropsychological Test of Automated Batteries (CANTAB™) Software (http://www.cambridgecognition.com/)
	Controlled Oral Word Association	Tombaugh, T. N., & Hubiey, A. M. (1997). The 60-item Boston Naming Test: Norms for cognitively intact adults aged 25 to 88 years. *Journal of Clinical and Experimental Neuropsychology, 19*, 922-932. doi:10.1080/01688639708403773
Attention / Working Memory	Letter-Number Sequencing	Wechsler, D. (2008). Wechsler Adult Intelligence Scale–Fourth Edition (WAIS–IV). *San Antonio, TX: NCS Pearson*
	Stroop (Colour-Word) Test	Fisher, L. M., Freed, D. M., & Corkin, S. (1990). Stroop Color-Word Test performance in patients with Alzheimer's disease. *Journal of Clinical and Experimental Neuropsychology, 12*, 745-758. doi:10.1080/01688639008401016
Memory	Hopkins Verbal Learning Test – Revised	Brandt, J., & Benedict, R. H. (2001). *Hopkins Verbal Learning Test-Revised: Professional Manual*. Lutz, FL: Psychological Assessment Resources.
		Hester, R. L., Kinsella, G. J., Ong, B., & Turner, M. (2004). Hopkins verbal learning test: Normative data for older Australian adults. *Australian Psychologist, 39*, 251-255. doi:10.1080/00050060412331295063
	Paragraph Recall	Wilson, B., Cockburn, J., Baddeley, A., & Hiorns, R. (1989). The development and validation of a test battery for detecting and monitoring everyday memory problems. *Journal of Clinical and Experimental Neuropsychology, 11*, 855-870. doi:10.1080/01688638908400940

		Strauss, E., Sherman, E., & Spreen, O. (2006). *A compendium of neuropsychological tests: Administration, norms, and commentary*. UK: Oxford University Press.
Visuospatial	Judgement of Line Orientation	Glamser, F. D., & Turner, R. W. (1995). Youth sport participation and associated sex differences on a measure of spatial ability. *Perceptual and motor skills, 81*, 1099-1105. doi:10.2466/pms.1995.81.3f.1099
		Ska, B., Poissant, A., & Joanette, Y. (1990). Line orientation judgment in normal elderly and subjects with dementia of Alzheimer's type. *Journal of Clinical and Experimental Neuropsychology, 12*, 695-702. doi:10.1080/01688639008401012
	Hooper Visual Organisation Test	Tamkin, A. S., & Jacobsen, R. (1984). Age- related norms for the Hooper Visual Organization Test. *Journal of clinical psychology, 40*, 1459-1463. doi:10.1002/1097-4679(198411)40:6<1459::AID-JCLP2270400633>3.0.CO;2-3
Language	Boston Naming Test – Short	Fastenau, P. S., Denburg, N. L., & Mauer, B. A. (1998). Parallel short forms for the Boston Naming Test: Psychometric properties and norms for older adults. *Journal of Clinical and Experimental Neuropsychology, 20*, 828-834. doi:10.1076/jcen.20.6.828.1105
	Similarities	Wechsler, D. (2008). Wechsler Adult Intelligence Scale–Fourth Edition (WAIS–IV). *San Antonio, TX: NCS Pearson*.
